# CD24 identifies nucleus pulposus progenitors/notochordal cells for disc regeneration

**DOI:** 10.1186/s13036-018-0129-0

**Published:** 2018-12-22

**Authors:** Zhuochao Liu, Zhiyong Zheng, Jin Qi, Jun Wang, Qi Zhou, Fangqiong Hu, Jing Liang, Changwei Li, Weibin Zhang, Xingkai Zhang

**Affiliations:** 1 Department of Orthopedics, Ruijin Hospital, Shanghai Jiaotong University School of Medicine, 197 Ruijin Er Road, Shanghai, 200025 China; 2 Shanghai Key Laboratory for Prevention and Treatment of Bone and Joint Diseases with Integrated Chinese-Western Medicine, Shanghai Institute of Traumatology and Orthopedics, Ruijin Hospital, Shanghai Jiaotong University School of Medicine, Shanghai, China; 3 Department of Orthopedics, Affiliated Hospital of Shandong University of Traditional Medicine, Jinan, China

**Keywords:** CD24, Nucleus pulposus progenitors, Notochordal cells, Disc regeneration

## Abstract

**Background:**

Cell-based therapy by transplantation of nucleus pulposus (NP) progenitor/notochordal cells has been proposed as a promising way to halt and reverse the progression of disc degeneration. Although some studies have provided a broad panel of potential markers associated with the phenotype of notochordal cells, suitability of these markers for isolation of notochordal cells for the treatment of disc degeneration is unclear.

**Results:**

Here, we found that the number of CD24-positive NP cells significantly decreased with increasing severity of disc degeneration. In addition, CD24-positive NP cells were shown to maintain their multipotent differentiation and self-renewal potential in vitro and to abundantly express brachyury, SHH, and GLUT-1, suggesting that CD24-positive NP cells are the progenitor/notochordal cells in the NP. Moreover, our in vivo experiments revealed that transplantation of CD24-positive NP cells enables the recovery of degenerate discs, as evidenced by increased disc height, restored magnetic resonance imaging T2-weighted signal intensity, and NP structure. In terms of the mechanism, HIF-1α–Notch1 pathway activation was essential for the maintenance of CD24-positive NP cells.

**Conclusion:**

Our studies identify that CD24-positive NP cells are the resident progenitor/notochordal cells in disc regeneration and elucidate a crucial role of HIF-1α–Notch1 pathway in the phenotypic maintenance of CD24-positive NP cells.

## Introduction

Disc degeneration is a ubiquitous disease in the aging adult population and is strongly implicated as a cause of lower back pain, a crippling condition that affects ~ 85% of all people at some point in their lifetime [1, 2]. The earliest manifestations of disc degeneration typically occur in the nucleus pulposus (NP), where reduced proteoglycan content compromises mechanical function leading to progressive structural deterioration of the entire intervertebral joint [3]. Although several risk factors, such as age, smoking, obesity, occupation, posture, and genetic inheritance have been reported to be related to disc degeneration, the molecular mechanisms involved in this degenerative process are not fully understood, and current therapies are mainly directed at managing symptoms without maintaining or restoring native disc structure or biomechanical function [3, 4]. A key focus of current research efforts is to develop new biological treatment strategies that can both address symptoms and regenerate native disc tissue.

An attractive strategy with great promise for long-term reconstitution of healthy disc tissue is cell-based regeneration, via which cells, alone or together with biomaterials, may be implanted into the NP to both repopulate and to stimulate native cells to produce a healthier extracellular matrix (ECM) [5]. Autologous NP cell implantation appears to be a good solution to repopulate the disc, and animal studies have shown that it can retard disc degeneration [6, 7]. Notochordal cells, which act as embryonic precursors to all cells found within the NP of a mature intervertebral disc (IVD), are present in the early stages of NP development, and are retained in the adult NP [8, 9]. In particular, loss of notochord cells has been correlated with the pathogenesis of disc degeneration and consequently, it has been hypothesized that regeneration of the disc could be mediated by notochord cells transplantation [10].

Cells with the typical notochordal morphology disappear from the disc within the first decade of life. Nonetheless, it can be difficult to distinguish notochordal from non-notochordal disc cells purely on the basis of morphology [9]. Therefore, harvesting notochordal cells with reference to a specific marker may be an ideal method. Some studies have provided a broad panel of potential markers associated with the phenotype of notochordal cells, highlighting cytokeratin 8 (CK-8), CK-18, CK-19, CD24, galectin (GAL3), and brachyury, which are expressed in a fetal notochordal immature NP as well as adult NP [9, 11]. To date, however, their suitability as unique markers for notochordal-cell isolation for direct therapeutic use in disc regeneration has not been evaluated.

Given that notochordal-cell implantation has been proposed as a promising cell-based therapeutic method for repairing disc degeneration, and CD24 has been proven to be a specific marker of notochordal cells, we set out to investigate the suitability of CD24 for notochordal-cell isolation and to determine whether CD24-positive NP cells have significant functional relevance to disc degeneration. Our findings uncovered a vital protective role of CD24-positive NP cells against disc degeneration and revealed that HIF-1α-NOTCH1 pathway activation is essential for the maintenance of CD24-positive notochordal cells.

## Results

### The number of CD24-positive NP cells decreased with the severity of disc degeneration

**Fig. 1:**
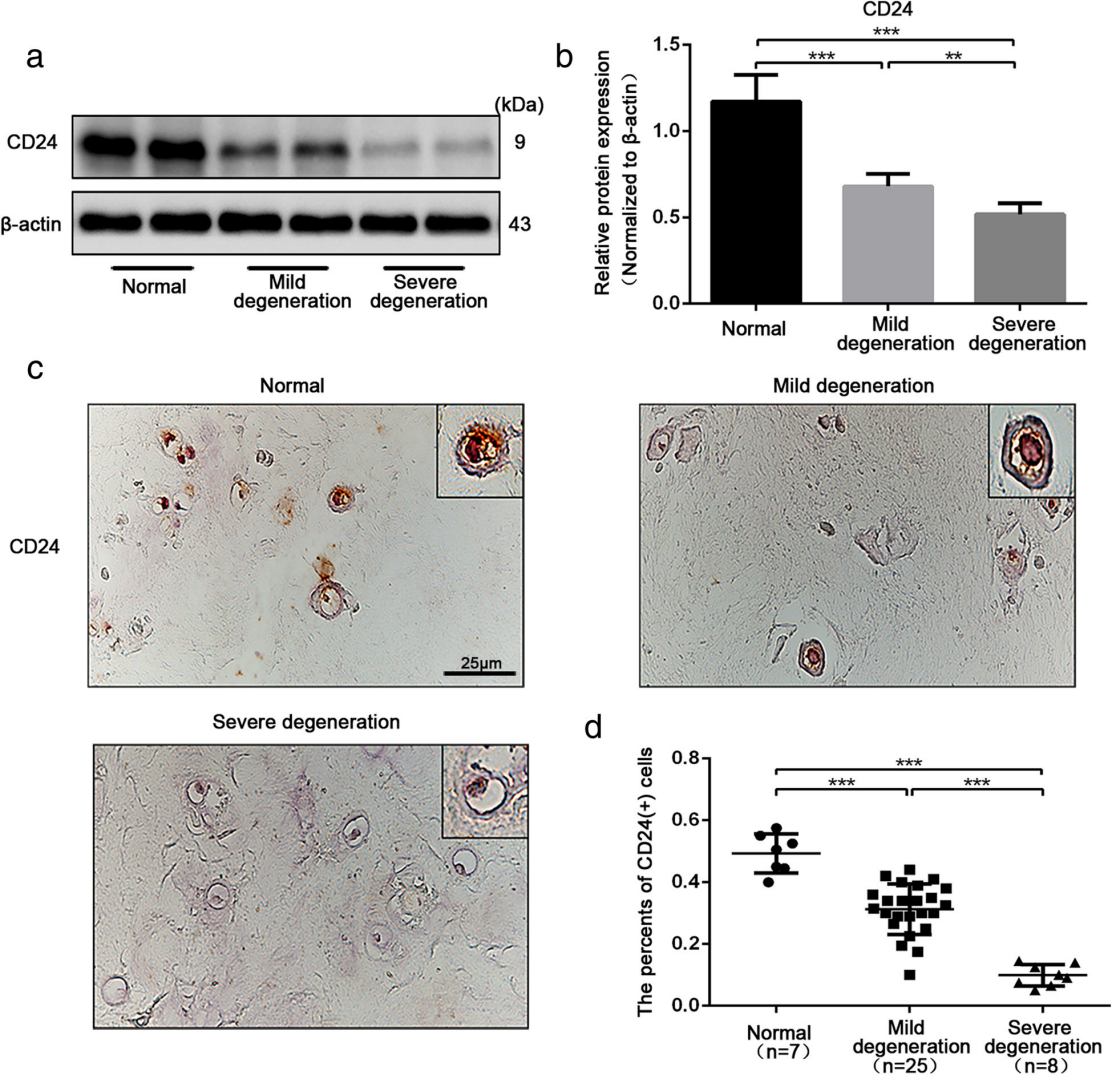
CD24^+^ NP cells decreased with the severity of disc degeneration. **a**-**b** Western blot analysis of CD24 expression in NP tissues with non, moderate and severe degeneration. **c**-**d** Immunohistochemical staining analysis of CD24 expression in NP tissues with non, moderate and severe degeneration. Scale bar represent 25 μm. **P < 0.01, ***P < 0.001. *P*-values were analyzed by one-way ANOVA

To investigate the clinical relevance of CD24-positive NP cells for disc degeneration, we first measured the abundance of CD24-positive cells in human NP tissues isolated from degenerated IVDs. Western blot analysis showed sustained CD24 expression in normal NP tissues (Fig. 1a-b). By contrast, with increasing severity of disc degeneration, CD24 expression decreased dramatically (Fig. 1a-b). The immunohistochemical analysis confirmed that the number of CD24-positive cells in NP tissues decreased significantly with increased degeneration severity (Fig. 1c-d). Taken together, these results indicated that CD24-positive cells in NP showed a negative relation to disc degeneration.

### CD24-positive cells are the resident progenitor/notochordal cells in the NP

**Fig. 2:**
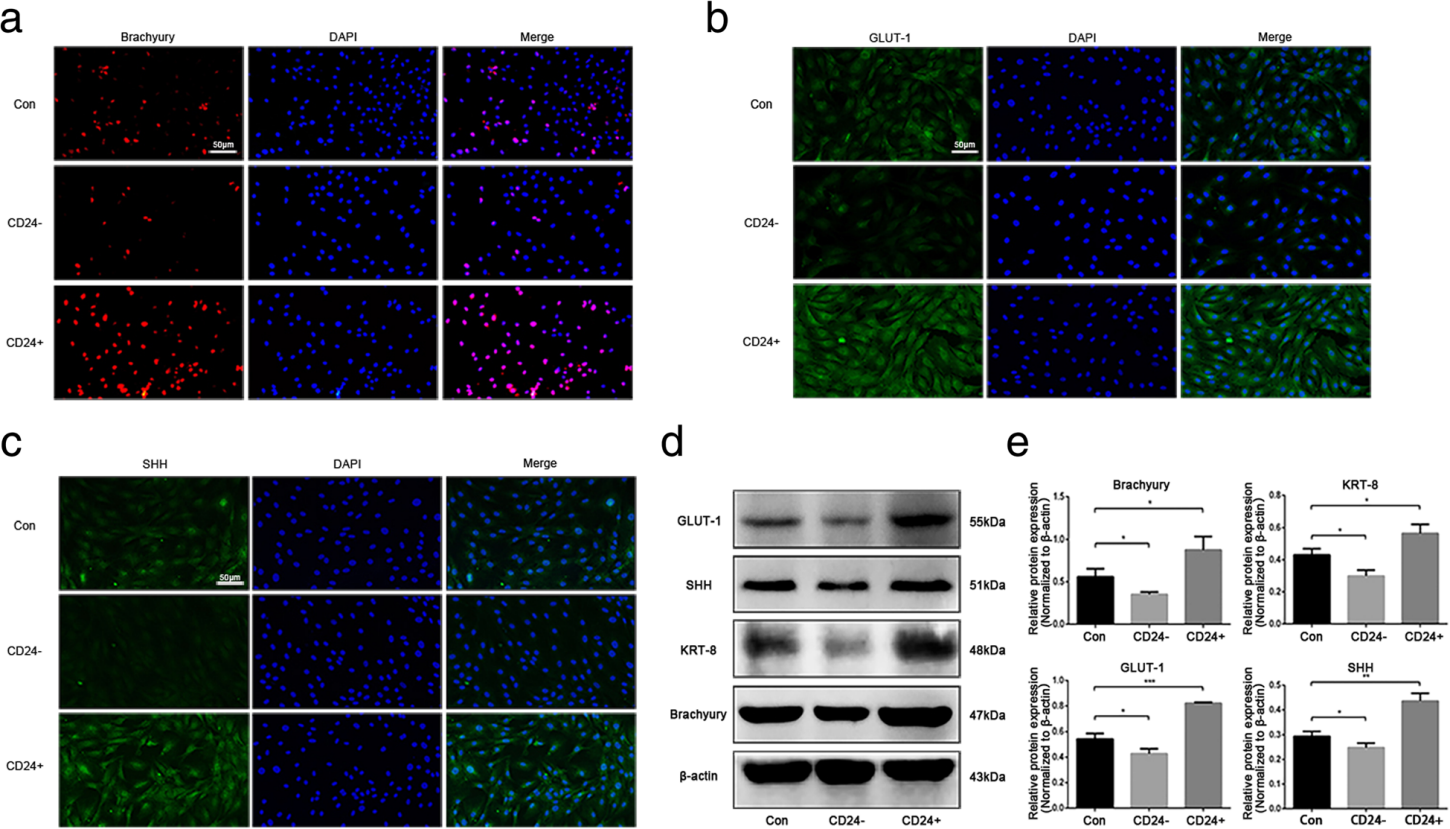
CD24^+^ NP cells express a higher level of notochordal/immature NP cell marker. **a**-**c** Immunofluorescence staining analysis of brachyury, SHH and GLUT-1 in CD24^**+**^, CD24^−^ and unsorted NP cells. **d**-**e** Western blot analysis of KRT8, brachyury, SHH and GLUT-1 expression in CD24^**+**^, CD24^−^ and unsorted NP cells. *P < 0.05, **P < 0.01, ***P < 0.001. *P*-values were analyzed by t one-way ANOVA. Scale bars represent 50 μm

**Fig. 3:**
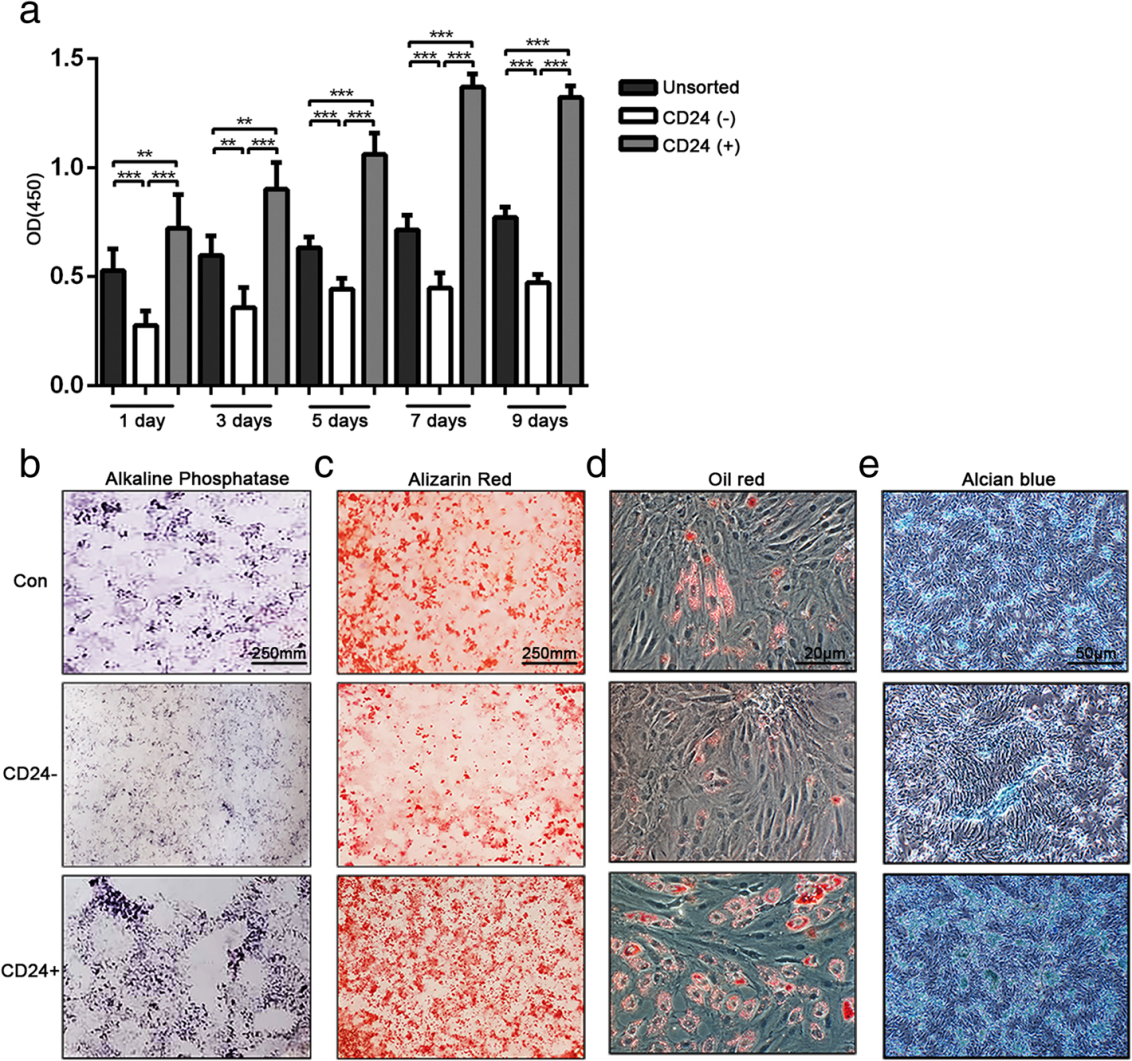
CD24^+^ NP cells exhibit a higher proliferation rate and higher osteogenic/adipogenic/chondrogenic differentiation ability than CD24^−^ and unsorted NP cells in vitro. **a** The proliferative activity of CD24^**+**^, CD24^−^ and unsorted NP cells detected by CCK8. **b**-**c** Osteogenic differentiation of CD24^**+**^, CD24^−^ and unsorted NP cells analysed by alkaline phosphatase (**b**) and alizarin red staining (**c**). **d** Adipogenic differentiation of CD24^**+**^, CD24^−^ and unsorted NP cells analysed by oil red O staining. **e** Chondrogenic differentiation of CD24^**+**^, CD24^−^ and unsorted NP cells analysed by alcian blue staining. **P < 0.01, ***P < 0.001. *P*-values were analyzed by one-way ANOVA

**Fig. 4:**
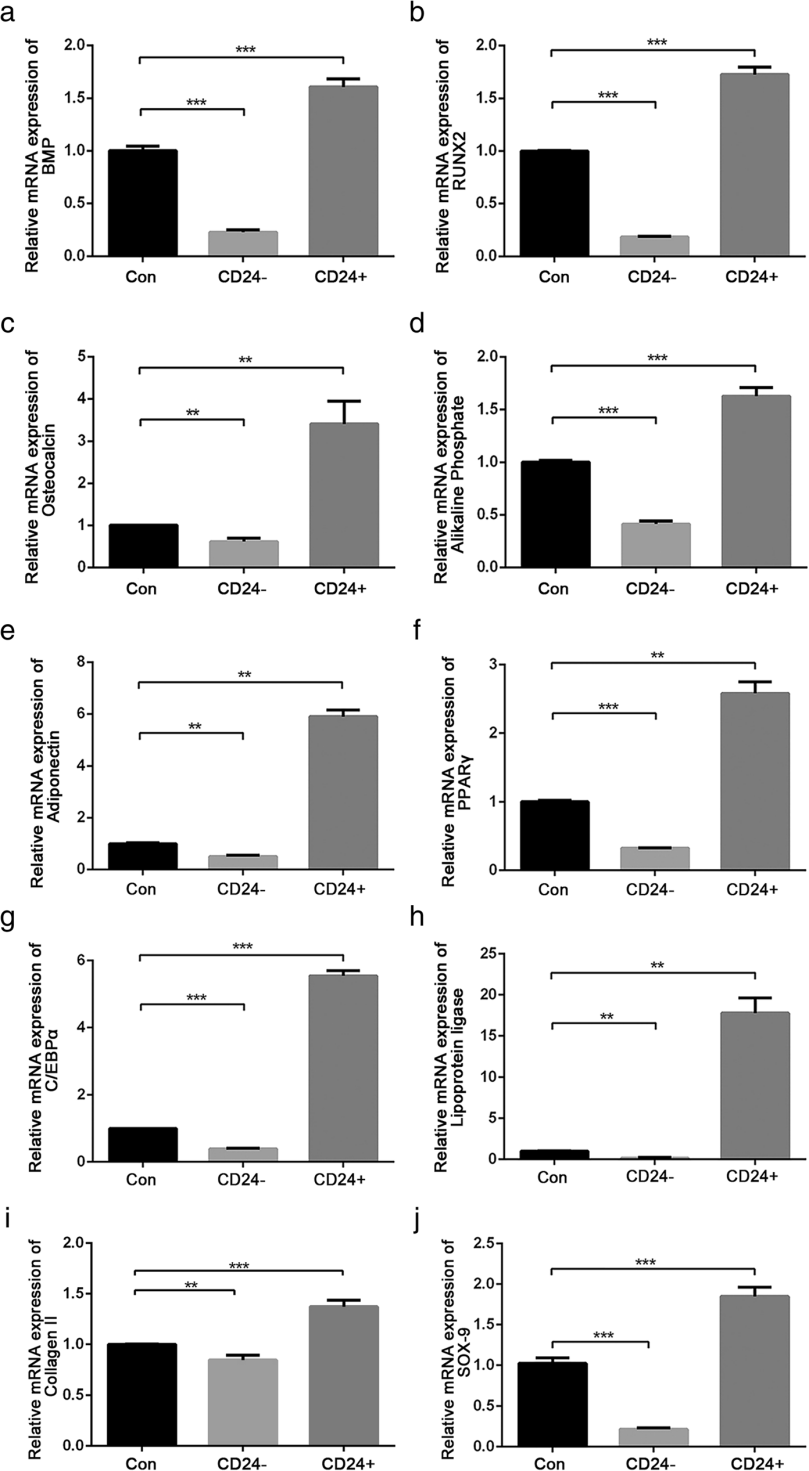
Osteogenic/adipogenic/chondrogenic differentiation related genes expression in CD24^+^ /CD24^−^ and unsorted NP cells after 3 weeks of differentiation induction. **a**-**d** Quantification analysis of BMP2 (**a**), RUNX2 (**b**), osteocalcin (**c**) and alkaline phosphate (**d**) expression in CD24^**+**^, CD24^−^ and unsorted NP cells after 3 weeks of osteogenic differentiation induction. **e**-**h** Quantification analysis of adiponectin (**e**), PPAR-γ (**f**), C/EBPα (**g**) and lipoprotein ligase (**h**) expression in CD24^**+**^, CD24^−^ and unsorted NP cells after 3 weeks of adipogenic differentiation induction. **i**-**j** Quantification analysis of COL II (**i**) and SOX-9 (**j**) expression in CD24^**+**^, CD24^−^ and unsorted NP cells after 3 weeks of chondrogenic differentiation induction. **P < 0.01, ***P < 0.001. *P*-values were analyzed by one-way ANOVA

To delineate the potential application of CD24-positive NP cells to disc degeneration repair, first, the phenotypes of CD24-positive NP cells were determined. CD24-positive and CD24-negative NP cells were isolated from rat NP tissues by fluorescence activated cell sorting (FACS). The immunofluorescent and western blot analyses showed that CD24-positive NP cells abundantly expressed brachyury, SHH, and GLUT-1 (Fig. 2a-e), which are proposed to be potential markers of notochordal/immature NP cells. Because notochordal cells are believed to be progenitor cells of the NP [12], we next evaluated the stemness of CD24-positive NP cells. The results showed that CD24-positive NP cells had a much higher proliferative activity than did CD24-negative NP cells (Fig. 3a). After 3 weeks of induction in three differentiation media designed for osteogenic differentiation, adipogenic differentiation, and chondrogenesis, formation of mineralized calcium nodules (Fig. 3b-c), lipid vacuoles (Fig. 3d), and proteoglycans (Fig. 3e), respectively, was found to be greater in CD24-positive NP cells than in CD24-negative and unsorted cells. In addition, quantification of the expression of osteogenic-differentiation–related genes, such as *bone morphogenetic protein 2* (*BMP2*) (Fig. 4a), *runt-related transcription factor 2* (*RUNX2*) (Fig. 4b), osteocalcin (Fig. 4c), and alkaline phosphatase (*ALP*) (Fig. 4d); of adipogenic-differentiation–related genes, such as *adiponectin* (Fig. 4e), (*peroxisome proliferator-activated receptor -γ*) *PPAR-γ* (Fig. 4f), *CCAAT/enhancer binding protein α* (*C/EBPα*) (Fig. 4g) and *lipoprotein lipase* (*LPL*) (Fig. 4h); and the expression of chondrogenic-differentiation–related genes, such as *collagen* II (*COL* II) (Fig. 4i) and *SOX-9* (Fig. 4j), revealed that CD24-positive NP cells confer an advantage over CD24-negative NP cells and unsorted NP cells in osteogenic, adipogenic, and chondrogenic differentiation. Taken together, these data showed that CD24-positive NP cells are the resident progenitor/notochordal cells in NP.

### Transplantation of CD24-positive NP cells ameliorates disc degeneration

**Fig. 5:**
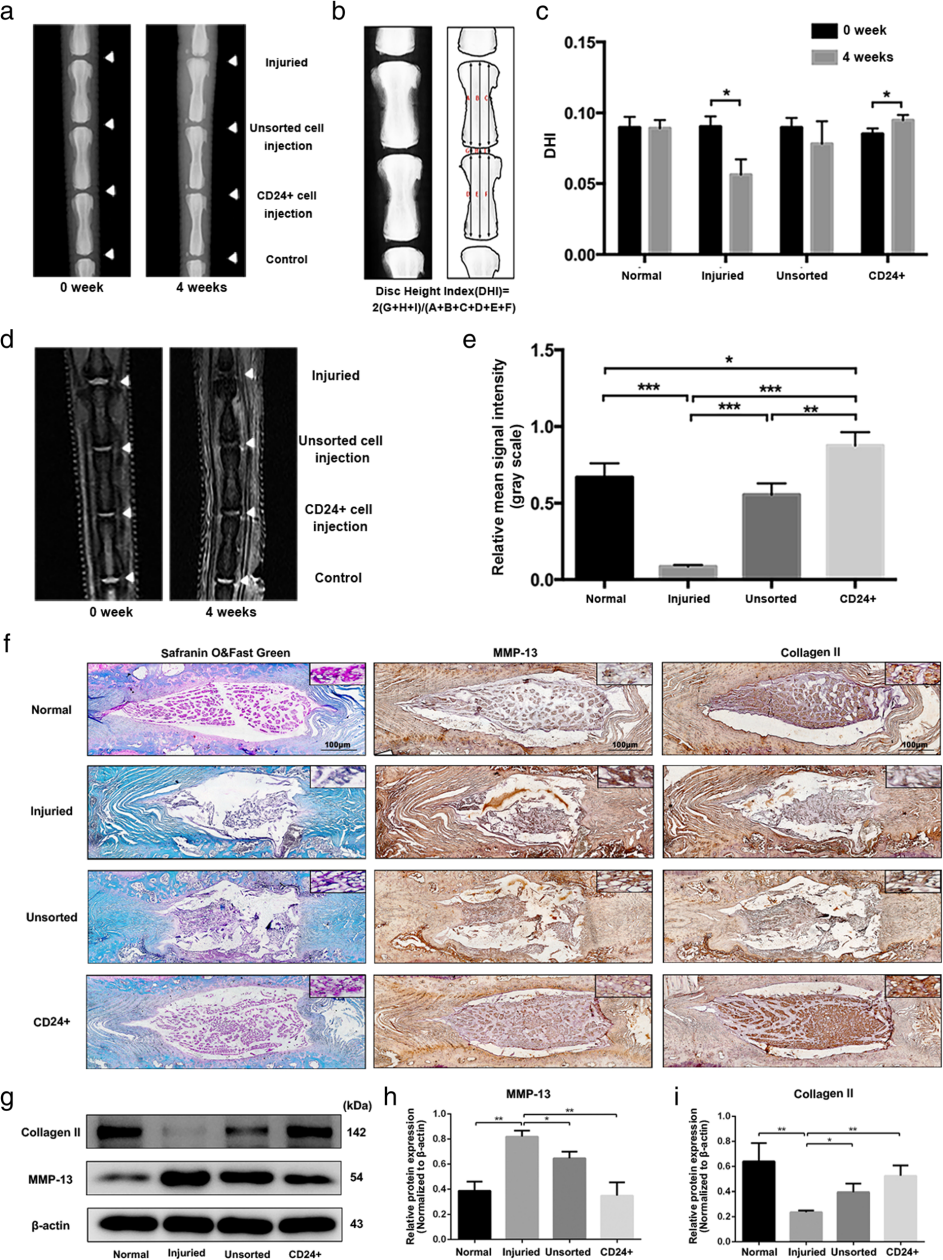
CD24^+^ NP cells transplantation ameliorates disc degeneration. **a** lateral X-ray images at 0 and 4 weeks after CD24^**+**^ and unsorted NP cells transplantation into the NP tissue punctured with needle. **b**-**c** Quantification of disc height at 0 and 4 weeks after CD24^**+**^ and unsorted NP cells transplantation into the NP tissue punctured with needle. **d**-**e** Midsagittal T2-weighted images at 0 and 4 weeks after CD24^**+**^ and unsorted NP cells transplantation into the NP tissue punctured with needle. **f** Histological analysis of intervertebral discs by Safranin O/Fast Green staining, immunohistochemical staining of collagen II and MMP13 in IVDs. Scale bars represent 100 μm. **g**-**i** Western blot analysis of collagen II and MMP13 in IVDs. *P < 0.05, **P < 0.01, ***P < 0.001. *P*-values were analyzed by two-tailed *t* tests in (**c**) and one-way ANOVA in (**e**-**i**)

Having established the notochordal/progenitor properties of CD24-positive NP cells, we next evaluated the therapeutic effect of CD24-positive NP cells on disc degeneration. The NP tissue of the rat lumbar IVD was punctured with a 21-gauge needle to induce disc degeneration [13] and was injected with PBS, unsorted NP cells, or CD24-positive NP cells. At 4 weeks after the surgery, we performed X-ray imaging and MRI analyses to assess the degeneration of the IVD. The X-ray analysis suggested that the transplantation of CD24-positive NP cells significantly reversed the disc height reduction induced by the puncture injury (Fig. 5a-c). No significant differences were detected between groups “surgery” and “unsorted NP cell injection” (Fig. 5a-c). Subsequent quantitative analysis of disc degeneration severity by MRI indicated a strong increase in the gray value of discs under the influence of transplantation of CD24-positive NP cells, observed as more hypointense signals in the midsagittal T2-weighted images (Fig. 5d-e). Safranin O/Fast Green staining revealed that puncture injury decreased the number of NP cells in the NP and interrupted the boundary between the NP and annulus fibrosus (AF), and that cracks were present in the NP. Nonetheless, in the CD24-positive NP cell transplantation group, the loss of proteoglycan induced by puncture injury was restored, most of the NP content was well preserved and the boundary between the NP and AF was interrupted minimally (Fig. 5f). In addition, the immunohistochemical staining as well as western blot analysis of collagen II and MMP13 suggested that CD24-positive NP cells transplantation obviously reduced the loss of collagen II, whereas dampens the increase of MMP13 expression in the degenerated IVDs (Fig. 5f-i). Taken together, these data proved that transplantation of CD24-positive NP cells alleviated disc degeneration.

### HIF-1α–NOTCH1 pathway activation is essential for the maintenance of CD24-positive NP cells

**Fig. 6:**
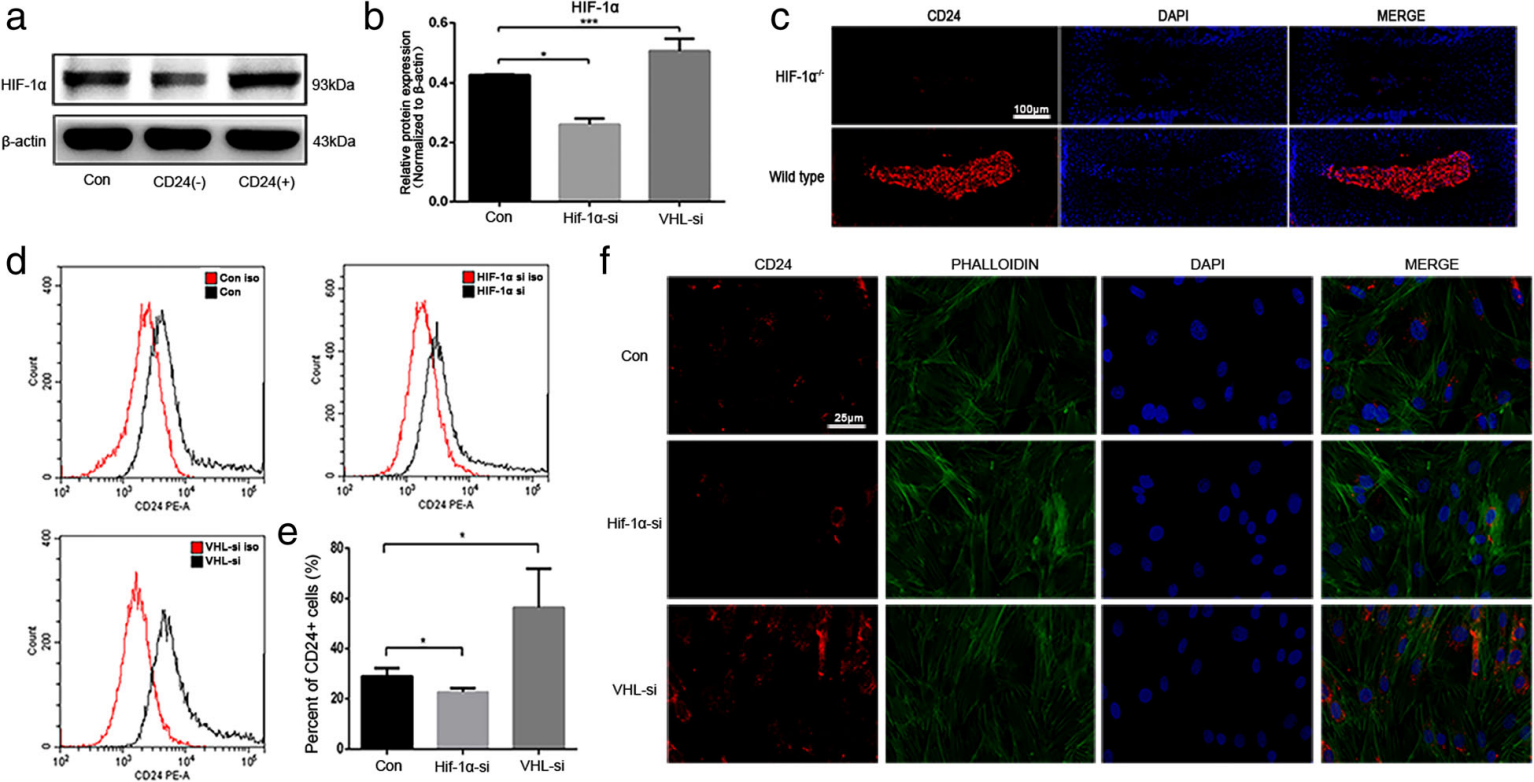
HIF-1α pathway activation is crucial for CD24^+^ NP cells maintenance. **a**-**b** Western blot analysis of HIF-1α expression in CD24^+^, CD24^−^ and unsorted NP cells. **c** Immunofluorescence staining analysis of CD24 in WT and NP-specific HIF-1α knock out mice, scale bars represent 100 μm. **d**-**e** Fluorescence activated cell sorting analysis of the percentage of CD24^**+**^ NP cells after *Hif-1α* or *Vhl* knock down. **f** Immunofluorescence staining analysis of CD24 in NP cells after *Hif-1α* or *Vhl* knock down, scale bars represent 25 μm. *P < 0.05, ***P < 0.001. *P*-values were analyzed by one-way ANOVA

Having established the progenitor properties and having delineated the protective effect of CD24-positive NP cells against disc degeneration, we next sought to investigate the underlying mechanisms that regulate differentiation of CD24-positive NP cells. The NP is an avascular tissue in a hypoxic environment, and our previous studies have revealed that hypoxia-inducible factor-1α (HIF-1α) performs an important function in NP cell survival and homeostasis of the ECM [14, 15]. Furthermore, our present results revealed that CD24-positive NP cells had a much higher level of HIF-1α expression than did CD24-negative NP cells and unsorted NP cells (Fig. 6a-b). Therefore, we hypothesized that HIF-1α might be a pivotal contributor to the maintenance of CD24-positive NP cells. To test this hypothesis, we first compared CD24 expression in the NPs between WT and NP-specific HIF-1α-deficient (NP-HIF-1α knockout) mice. The immunofluorescence analysis revealed that in contrast to the substantial number of CD24-positive cells in the NP of WT mice, HIF-1α deficiency led to disappearance of CD24-positive NP cells (below the detection limit) (Fig. 6c). Likewise, the in vitro results showed that the *Hif-1α* knockdown decreased the percentage of CD24-positive cells (Fig. 6d-e); however, overexpression of *Hif-1α* via *von Hippel-Lindau* (*Vhl*) deletion resulted in an increased proportion of CD24-positive cells (Fig. 6f).

**Fig. 7:**
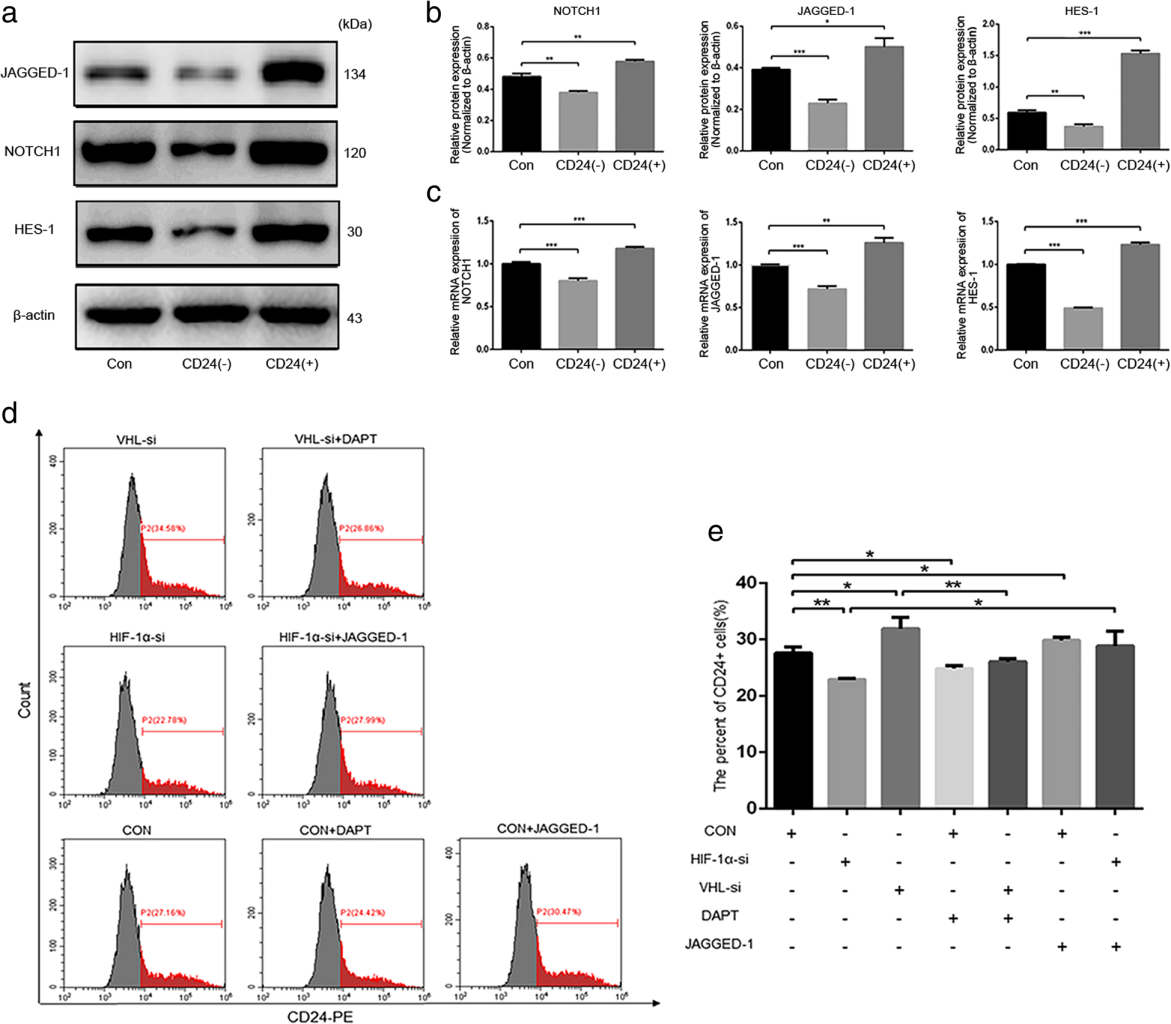
HIF-1α-NOTCH1 pathway activation is essential for CD24^+^ NP cells maintenance. **a**-**b** Western blot analysis of JAGGED-1, NOTCH1 and HES-1 expression in CD24^+^, CD24^−^ and unsorted NP cells. (c) Quantification analysis of JAGGED-1, NOTCH1 and HES-1 mRNA expression in CD24^+^, CD24^−^ and unsorted NP cells. **d**-**e** Fluorescence activated cell sorting analysis of the percentage of CD24^**+**^ NP cells after *Hif-1α* or *Vhl* knock down with or without DAPT and JAGGED-1 stimulation. *P < 0.05, **P < 0.01, ***P < 0.001. *P*-values were analyzed by one-way ANOVA

Given that NOTCH1 signaling is critical for the maintenance of NP cell proliferation and ECM metabolism in the hypoxic niche of a disc [15], and our present results revealed that CD24-positive NP cells have a higher level of NOTCH1, the NOTCH1 ligand JAGGED1, and its target gene *hairy and enhancer of split-1* (*HES1*) than CD24-negative and unsorted NP cells (Fig. 7a-c). In our chasing experiments, we sought to detect the involvement of the NOTCH1 pathway in the maintenance of CD24-positive NP cells. The FACS results suggested that activation of the NOTCH1 pathway with JAGGED-1 significantly restored the proportion of CD24-positive NP cells decreased by the HIF-1α knockdown (Fig. 7d-e). In contrast, NOTCH1 inhibitor DAPT obviously attenuated the CD24 upregulation caused by *Vhl* deletion (Fig. 7). Taken together, these data showed that HIF-1α–NOTCH1 pathway activation is essential for the maintenance of CD24-positive NP cells.

## Discussion

Cell-based therapies involving injection of IVD cells, chondrocytes, or stem cells have gained significant research insights and progressed to clinical trials for the treatment of spinal disorders [16]. Progenitor cells do have an advantage over terminally differentiated cells in that the progenitors maintain their multipotent differentiation and self-renewal potential in vivo and in vitro under appropriate conditions. Furthermore, these cells play an important role in the development and homeostasis of IVD tissue [17]. Notochordal cells have been proposed as embryonic precursors to all cells found within the NP of a mature IVD, and undergo terminal differentiation to give rise to chondrocyte-like cells [8]. Furthermore, the loss of notochordal cells is associated with the onset of disc degeneration, suggesting that these cells are required for the maintenance of the NP [10]. Therefore, identifying the NP-derived progenitor cells/notochordal cells and transplanting them into the NP may be a promising way to halt and reverse the progression of disc degeneration.

In humans, the NP is composed of large vacuolated notochordal cells in the fetus, but soon after birth, becomes populated by smaller, chondrocyte-like cells. Although animal studies indicate that notochord-derived cells persist in the adult NP, the ontogeny of the adult human NP cell population is still unclear [11]. Thus, identification of unique notochordal markers is required. In the present study, we found that the proportion of CD24-positive NP cells significantly decreased with increasing severity of disc degeneration. Although no complete set of markers of notochordal cells has yet been identified, the findings of this study suggest that CD24-positive NP cells may be the resident NP progenitor/notochordal cells, as demonstrated by the presence of multiple notochordal-cell–associated markers, including brachyury, SHH, and GLUT-1. In addition, CD24-positive NP cells were shown to maintain the self-renewal potential and undergo directed in vitro differentiation, producing cells that are morphologically and phenotypically similar to chondrocyte-like NP cells. Moreover, our in vivo experiments revealed that transplantation of CD24-positive NP cells enables the recovery of degenerate discs, as evidenced by increased disc height and restored MRI T2-weighted signal intensity and NP structure, as well as increased expression of proteoglycan and collagen II in NPs, which indicated that the transplanted CD24-postive NP cells might differentiate into chondrocyte-like cells. However, to test this hypothesis, the labeled CD24-postive NP cells are needed to be employed in the further studies. Therefore, CD24 might be a suitable marker of NP progenitor/notochordal cells; moreover, transplantation of CD24-positive NP cells might exert a promising therapeutic effect on disc degeneration.

The NP is an avascular tissue in a hypoxic environment, and it has been demonstrated that NP cells maintain their biosynthetic activities via constitutive expression of HIF-1α [18]. Our previous study has revealed that HIF-1α is a pivotal mediator of the development of the NP and has a prosurvival function in NP cells; this is because we find that there are many more cell deaths in the NP of NP-specifically HIF-1α deficient mice at 3 days, 6 weeks, and 12 weeks after birth [14]. Furthermore, our present study suggests that there is much less HIF-1α expression in CD24-negative NP cells than in CD24-positive NP cells. On this basis, we hypothesized that HIF-1α is essential for the maintenance of CD24 NP cells. Our results showed that CD24-positive NP cells were nearly undetectable in NPs of NP-specifically HIF-1α–deficient mice. Moreover, our in vitro results indicated that HIF-1α deletion decreased whereas HIF-1α overexpression via *VHL* knockdown increased the percentage of CD24-positive NP cells. Therefore, our results mean that HIF-1α is a crucial mediator in the maintenance of CD24-positive NP cells.

Besides promoting the survival of NP cells, HIF-1α plays an important part in ECM synthesis [19]. Our previous study indicates that NOTCH1 works as a downstream pathway of HIF-1 in ECM metabolism as well as in the maintenance of NP cells’ proliferation [15]. Therefore, we advanced the hypothesis that NOTCH1 might also be essential for the maintenance of CD24-positive NP cells. Our results showed that activation of NOTCH1 with JAGGED-1 rescued the percentage of CD24-positive NP cells decreased by HIF-1α deletion, whereas inhibition of NOTCH1 with DAPT attenuated the increase in the percentage of CD24-positive NP cells after the VHL knockdown. Therefore, our data revealed that NOTCH1 is a crucial downstream mediator of HIF-1α action for the maintenance of CD24-positive NP cells.

## Conclusion

**Fig. 8:**
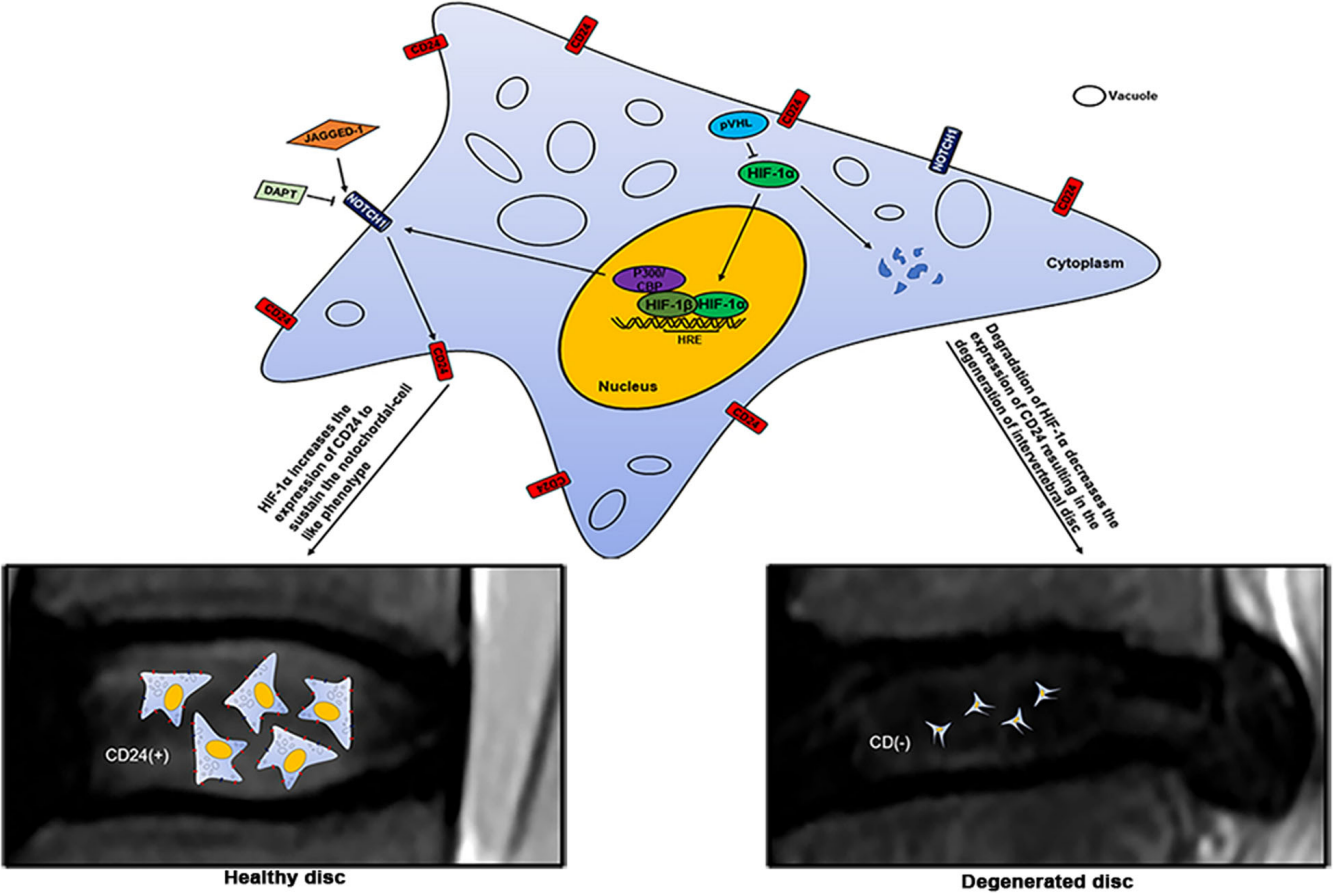
The schematic graph reflects HIF-1α-NOTCH1 in the maintenance of CD24^+^ NP cells. HIF-1α-NOTCH1 pathway is essential for the maintenance of CD24^+^ NP cells, and which drives disc regeneration

The identification of CD24-positive NP cells as the resident progenitor cells/notochordal cells in disc regeneration as well as elucidation of the crucial role of the HIF-1α–NOTCH1 pathway in the phenotypic maintenance of CD24-positive NP cells provides new insights into the beneficial effect of NP progenitor/notochordal cells for the treatment of disc degeneration (Fig. 8).

## Materials and methods

### Human tissue collection and grading

In accordance with the Institutional Review Board guidelines of Ruijin Hospital affiliated with Shanghai Jiaotong University School of Medicine, all patients underwent Pfirrmann grading based on magnetic resonance imaging (MRI) results [20]. Human NP specimens were collected from 40 patients (Pfirrmann grades 1–5, mean age of 47.13 years, 23 males and 17 females) with IVD herniation, spinal stenosis, or lumbar fracture. The Pfirrmann score of normal, mild degeneration, and severe degeneration groups is 1 or 2, 3 or 4, and 5, respectively. All the patients consented to the sample collection. In all, 40 samples from 40 patients were obtained; characteristics of the patients are summarized in Table 1.

**Table 1:** characteristics of 40 patients

Patient no.	Sex	Age	Diagnosis	Sample level	Pfirrmann grade
1	male	70	intervertebral disc herniation	L4-L5	5
2	male	52	spondylolisthesis	L4-L5	5
3	female	46	intervertebral disc herniation	L5-S1	4
4	female	73	intervertebral disc herniation	L4-L5	4
5	male	21	intervertebral disc herniation	L5-S1	4
6	female	72	spinal stenosis	L4-L5	4
7	male	68	intervertebral disc herniation	L4-L5	4
8	male	67	intervertebral disc herniation	L4-L5	4
9	female	74	spinal stenosis	L4-L5	5
10	male	59	spinal stenosis	L3-L4	4
11	female	71	intervertebral disc herniation	L4-L5	5
12	male	33	intervertebral disc herniation	L5-S1	4
13	male	62	spinal stenosis	L5-S1	3
14	male	49	spinal stenosis	L4-L5	4
15	male	29	intervertebral disc herniation	L5-S1	4
16	female	56	spinal stenosis	L5-S1	4
17	female	42	intervertebral disc herniation	L5-S1	4
18	female	18	lumbar fracture	L1	2
19	male	23	intervertebral disc herniation	L5-S1	4
20	male	25	intervertebral disc herniation	L5-S1	3
21	male	28	lumbar fracture	L1	2
22	male	44	intervertebral disc herniation	L4-L5	4
23	male	50	intervertebral disc herniation	L4-L5	5
24	female	57	spinal stenosis	L5-S1	5
25	female	58	intervertebral disc herniation	L4-L5	3
26	male	33	intervertebral disc herniation	L5-S1	5
27	female	34	intervertebral disc herniation	L4-L5	4
28	male	43	spinal stenosis	L4-L5	3
29	male	40	ankylosing spondylitis	L3-L4	3
30	female	28	lumbar fracture	L1	2
31	male	23	lumbar fracture	L1	2
32	female	57	spinal stenosis	L4-L5	4
33	female	73	spinal stenosis	L4-L5	4
34	male	67	intervertebral disc herniation	L4-L5	5
35	male	59	intervertebral disc herniation	L3-L4	4
36	male	62	spinal stenosis	L4-L5	3
37	female	18	spondylolisthesis	L5-S1	2
38	male	41	lumbar fracture	L3	1
39	female	47	intervertebral disc herniation	L5-S1	3
40	female	13	spondylolisthesis	L5-S1	2

### Cell isolation and culture conditions

The experiment was approved by the animal experimentation committee of Ruijin Hospital, affiliated with Shanghai Jiaotong University School of Medicine NP cells were isolated from 6- to 8-week-old male Sprague–Dawley rats. Each rat was euthanized with sodium pentobarbital, and its lumbar spine (L1–L5) was removed aseptically. The gel-like NP was separated from an IVD using a dissecting microscope and digested with 0.25% trypsin for 30 min first, then treated with 0.1% type II collagenase (Gibco) for 4–6 h. The partially digested NP tissue was filtered through a 70 μm cell strainer (Falcon) and centrifuged at 500 x g for 5 min. The precipitate was maintained in Dulbecco’s modified Eagle medium-F12 (DMEM/F12, GIBCO) with 10% fetal bovine serum (FBS, Gibco) and a 1% penicillin–streptomycin solution (Gibco) in a humidified atmosphere containing 5% of CO_2_ at 37 °C. When NP cells reached confluence, the cells were digested with 0.25% trypsin-EDTA and subcultured in 10 cm dishes. To examine the effect of the NOTCH pathway, we used recombinant Human JAGGED-1(c-Fc) (10 μg/ml, 1277-JG, R&D Systems) to precoat a 12-well plate before NP cells were cultured in it, and DAPT (10 μM, 2634–10, R&D Systems) was added to the culture medium after transfection with small interfering RNA (siRNA).

### Fluorescence-activated cell sorting (FACS)

To isolate CD24-positive NP cells, the NP cells were resuspended in 100 μl of phosphate-buffered saline (PBS) containing 1% of FBS and were incubated with a mouse anti-rat CD24 antibody (551,133, BD Pharmingen) for 30 min at 4 °C. The incubation was performed for additional 30 min at 4 °C in the dark with an Alexa Fluor® 488–conjugated donkey anti-mouse IgG antibody (secondary antibody; A21202, Molecular Probes). After incubation, the NP cells were washed three times with PBS. Sorting was performed on MoFlo Astrios (Beckman, USA). We collected CD24-negative and -positive cells separately and cultured the sorted NP cells in a 6-well plate for the following experiments.

### Cell viability measurement

The Cell Counting Kit 8 (CCK-8, Dojindo, Japan) was used. The CD24-negative, CD24-positive, and unsorted NP cells were seeded at a density of 5 × 10^3^ cells/well in 96-well plates and incubated for 1, 3, 5, 7 and 9 days. Then, the medium of the NP cells was changed, and 10 μl of CCK-8 was added into each well. After incubation for 1 h at 37 °C, cell viability was quantified by measuring the absorbance at 450 nm on Infinite 200 pro (Tecan, Switzerland).

### Multilineage differentiation of NP cells

For osteogenic differentiation, CD24-negative, CD24-positive, and unsorted NP cells were plated at 5 × 10^3^ cells/well in a 6-well plate. When the NP cells reached 90% confluence, the medium was replaced with an osteogenic medium (DMEM/F12 supplemented with 10% FBS, 1% penicillin–streptomycin solution, 100 nM dexamethasone, 200 μM ascorbic acid, and 10 mM β-phosphoglycerol). The medium was changed every 3 days. After 21 days, the cells were harvested and washed with PBS. For alizarin red and alkaline phosphatase staining, the NP cells were fixed in 4% paraformaldehyde for 15 min, and then incubated with different reagents following manufacturers’ instructions.

For adipogenic induction, NP cells were seeded in a 6-well plate as mentioned above. When the NP cells reached 80% confluence, the medium was replaced with an adipogenic medium (DMEM/F12 supplemented with 10% FBS, 1% penicillin-streptomycin, 1 μM dexamethasone, 0.45 mM 3-isobutyl-1-methylxanthine, 50 μg/ml indomethacin, and 10 μg/ml insulin). The inducing medium was changed every 2 days, and next, the NP cells were incubated with a maintaining medium (DMEM/F12 supplemented with 10% of FBS, 1% of the penicillin–streptomycin solution, 10 μg/ml insulin) for 1 day. One cycle was composed of 3 days, and the whole process was sustained for 7 cycles. For oil-red-O staining, the NP cells were fixed in 4% paraformaldehyde for 15 min and then washed. The fixed NP cells were incubated with oil-red-O for 1 h at room temperature.

For chondrogenic differentiation, NP cells were seeded in a 6-well plate as mentioned above. When the cells reached 80% confluence, the medium was replaced with a chondrogenic medium (ITS+, 50 mg/ml ascorbic acid 2-phosphate, 40 mg/ml proline, 100 nM dexamethasone, 10 ng/ml TGFβ3, 100 mg/ml sodium pyruvate). After 21 days, the result of chondrogenic differentiation was evaluated by alcian blue staining and qRT-PCR.

### Transfection of siRNA

To verify the effects of Hif-1α and the notochordal-cell-like phenotype, siRNA technology was used according to the manufacturer’s instructions. Briefly, the NP cells seeded in 6-well plates (80% confluent) were transfected with a mixture of Lipofectamine 3000 (Thermo) and *Hif-1α* siRNA or *Vhl* siRNA (GenePharma, China) respectively. Twenty-four hours after transfection, the medium was replaced with DMEM/F12 supplemented with 10% of FBS and 1% of the penicillin–streptomycin solution.

### Flow-cytometric analysis

To confirm the relation between Hif-1α and the notochordal-cell-like phenotype, the control, Hif-1α knockdown, and Vhl knockdown NP cells were digested with 0.25% trypsin and resuspended in 100 μl of PBS. After that, we incubated NP cells with a phycoerythrin (PE)-conjugated anti-rat CD24 antibody (130–106-254, Miltenyi) for 30 min at 4 °C in the dark. Finally, the cells were washed three times and analyzed on Beckman CytoFlex S (Beckman, USA).

### Immunofluorescence analysis

Cell slides were washed with PBS before fixation with 4% paraformaldehyde three times. The cryosections and cell slides of control and Hif-1α knockout mice at the age of 2 weeks were pretreated with Triton X-100 for 15 min, incubated with anti-brachyury (ab209665, Abcam), anti-GLUT-1 (ab652, Abcam), anti-SHH (sc-365,112, Santa Cruz), anti-CD24 (ab64064, Abcam), and anti-CD24 antibodies (130–106-254, Miltenyi) at appropriate dilutions (according to the manufacturer’s instructions) at 4 °C overnight. Slides were then rinsed with PBST (PBS with 0.1% Tween-20) three times for 5 min and incubated with secondary antibodies: an Alexa Fluor® 488–conjugated goat anti-rabbit IgG antibody (A11008, Molecular Probes), Alexa Fluor ® 594–conjugated goat anti-rabbit IgG antibody (A11072, Molecular Probes), or Alexa Fluor® 488–conjugated donkey anti-mouse IgG antibody (A21202, Molecular probe) at 1:1000 dilutions for 1 h at 37 °C in the dark. The staining was visualized by means of a microscope (Olympus, Japan) equipped with a digital camera (Olympus, Japan).

### Western blot analysis

Cell samples were lysed in RIPA buffer on ice for 20 min. The lysed cells were collected and centrifuged at 14,000 x *g* to remove cell debris. Protein concentrations were determined with the BCA Protein Assay Kit (P0009, Beyotime). Each sample containing 30 μg of total protein was separated by SDS-PAGE in a 10% gel and transferred onto PVDF membranes (EMD Millipore Corporation, US). After blockage with 5% nonfat dry milk in Tris-buffered saline with 1‰ Tween (TBST), the membranes were incubated overnight at 4 °C with primary antibodies against brachyury (ab209665, Abcam), GLUT-1 (ab652, Abcam), SHH (sc-365,112, Santa Cruz), CD24 (ab64064, Abcam), KRT-8 (sc-8020, Santa Cruz), HIF-1α (NB100–105, Novus), NOTCH1 (4380, CST), JAGGED1 (ab109536, Abcam), HES1 (11,988, CST), and β-actin (CW0096S, CW Biotech). After three washes with TBST, the membrane was incubated with horseradish peroxidase–conjugated secondary antibodies (Jackson). The protein expression level was determined by densitometric analysis and was normalized to the level of β-actin.

### Quantitative RT-PCR analysis

mRNA expression of Hif-1α and Vhl, markers of notochordal cells (brachyury, SHH, KRT-8, KRT-18, and GLUT-1), and components of the NOTCH pathway (NOTCH1, JAGGED-1, and HES-1), and osteogenic markers (BMP-2, RUNX2, OC, and ALP), and adipogenic markers (adiponectin, PPAR-γ, CEBP/α, and LPL), and chondrogenic markers (COL II and SOX-9) was analyzed by qRT-PCR. Briefly, after total RNA was extracted from NP cells with the TRIzol Reagent (Invitrogen); total RNA was used to synthesize complementary DNA (cDNA) with a reverse transcription kit (Takara, Japan). The reaction system was composed of specific primers, cDNA, and the SYBR Green qPCR Mix (Takara, Japan). Primers (Table 2) were synthesized by a private company (Sangon, Biotech Co. Ltd., China). β-Actin served as the reference gene, and the expression of target genes was calculated as 2^—△△Ct^.

**Table 2:** Primer sequences for real time-PCR

Gene		Primer sequence(5′- 3′)
BRACHYURY	FORWARD	CTGCAGTACCGAGTGGATCA
	REVERSE	ACAGCTCGCTCTCTTCCAGA
GLUT-1	FORWARD	ACGGTGCTGCTGGTACTCTT
	REVERSE	TCAGGTGTCTTGTCGCTCTG
SHH	FORWARD	ATGAACCAGTGGCCTGGA
	REVERSE	CCTGTCAGACGTGGTGATGT
KRT-8	FORWARD	CAGGAGCTGATGAACGTCAA
	REVERSE	TTCGTGTGGATGCTCATGTT
KRT-18	FORWARD	GCTGAGACCACACTCTTGGAG
	REVERSE	TGTATCTGGCCTCCACTTCC
NOTCH1	FORWARD	TCGTGCTCCTGTTCTTTGTG
	REVERSE	TTCTCTCCGCTTCTTCTTGC
JAGGED-1	FORWARD	CTGAGGACTACGAGGGCAAG
	REVERSE	CCCTTCAGGAGTATCGTTGG
HES-1	FORWARD	CCCGTCTACCTCTCTCCTTG
	REVERSE	TCCCCTTTACTTGGCTTTCA
HIF-1α	FORWARD	TCAAGTCAGCAACGTGGAAG
	REVERSE	TTCACAAATCAGCACCAAGC
RUNX2	FORWARD	CCACCACTCACTACCACACG
	REVERSE	GGACGCTGACGAAGTACCAT
OSTEOCALCIN	FORWARD	CCTGACTGCATTCTGCCTCT
	REVERSE	AGGTAGCGCCGGAGTCTATT
BMP-2	FORWARD	GAAGCCAGGTGTCTCCAAGA
	REVERSE	GGATGTCCTTTACCGTCGTG
ALP	FORWARD	GACAAGAAGCCCTTCACAGC
	REVERSE	ACTGGGCCTGGTAGTTGTTG
ADIPONECTIN	FORWARD	ACCCAAGGAAACTTGTGCAG
	REVERSE	CGTCTCCCTTCTCTCCCTTC
C/EBPα	FORWARD	GCCAAGAAGTCGGTGGATAA
	REVERSE	AACACCTTCTGCTGCGTCTC
PPAR-γ	FORWARD	CGAGAAGGAGAAGCTGTTGG
	REVERSE	TCAGCGGGAAGGACTTTATG
LPL	FORWARD	CAGCTGGGCCTAACTTTGAG
	REVERSE	GGATCCCAATACTTCGACCA
SOX-9	FORWARD	TCAACGGCTCCAGCAAGAACAAG
	REVERSE	CTCCGCCTCCTCCACGAAGG
COL-II	FORWARD	CTCAAGTCGCTGAACAACCA
	REVERSE	GTCTCCGCTCTTCCACTCTG
β-ACTIN	FORWARD	ACGGGAATGTGAAGATGACC
	REVERSE	CTCTGACACCACAGGAGCAA

### Surgery

To eliminate individual differences, we performed the surgery and injected different kinds of NP cells into different segments of the same rat. Six Sprague–Dawley rats (6–8 weeks old, male) were chosen randomly. The rats were injected with sodium pentobarbital (30 mg/kg) intraperitoneally. Then, they were fixed on the board in a prone position, with their tail fully displayed in the surgical field of vision. The position of an IVD was located by X-ray imaging. The depth of puncture was controlled at 5 mm by a hemostat, which ensured that the needle would arrive at the middle of an IVD (the depth from skin to the center of the NP is about 5 mm). The needle (21G) was kept in place with rotation for 20 s and then by injection with 50 μl of PBS alone or PBS containing 1 × 10^5^ CD24-positive or unsorted NP cells separately. Four weeks after the surgery, the degree of degeneration was determined by X-ray imaging and MRI.

### Radiographic assessments

X-ray images of the rat caudal spine were captured preoperatively and 4 weeks after the puncture. When radiographs were taken, rat tails were laid straight, resting on a plastic plate. The change in IVD height was expressed as the disc height index (DHI) according to the method outlined by Mazuda [21]. Disc Height Index (DHI) = 2 × (DH1 + DH2 + DH3) ÷ (LB1 + LB2 + LB3 + UB1 + UB2 + UB3). UB and LB represent the length of the upper vertebrae body and the lower vertebrae body, respectively. The imaging of rat caudal vertebrae was also performed simultaneously by 7.0 T MRI (BioSpec 70/20 USR, Bruker). When an IVD degenerated, the water content decreased, and the IVD showed low intensity in a T2-weighted image. The IVDs in MRI images were thus quantitated by means of a gray scale.

### Histological staining

Six rats were euthanized by intraperitoneal injection of sodium pentobarbital at 4 weeks after the surgery. Their whole tail was removed. The skin, muscles, and ligaments were carefully removed from the tail, which was then fixed in 4% paraformaldehyde for 2 days and rinsed with PBS several times before immersion in a decalcification solution. After decalcification and dehydration, the caudal vertebrae were embedded into paraffin. Paraformaldehyde-fixed paraffin-embedded caudal vertebrae were cut in the sagittal position. The sections were then stained with hematoxylin and eosin.

### Immunohistochemistry

The sections were deparaffinized, rehydrated, and then processed with the Super Sensitive TM IHC Detection System kit (BD5001, Bioworld, USA). After blockage, the sections were incubated with an anti-CD24 antibody (ab199140, Abcam) overnight at 4 °C. These steps were carried out according to the vendor’s instructions. We randomly selected visual fields to count positive cells until a total of 200 NP cells were counted in each section; the total number of positive cells is expressed as a percentage of all cells.

### Statistical analysis

All data are present All data are representative of three independent experiments and are as mean ± SEM. We used two-tailed t tests to determine significances between two groups. We did analyses of multiple groups by one-way ANOVA with Bonferroni post test of GraphPad prism version 5. For all statistical tests, we considered *P* value < 0.05 to be statistically significant.

## Abbreviations

ALP: And alkaline phosphate
BMP2: Bone morphogenetic protein 2
C/EBPα: CCAAT/enhancer binding protein α
CD24^-^: CD24 negative
CD24^+^: CD24 positive
CK-8: Cytokeratin 8
COLII: Collagen II
FACS: Fluorescence activated cell sorting
GLUT-1: Glucose transporter type I
HES-1: Hairy and enhancer of split-1
HIF-1α: Hypoxia-inducible factor-1α
IVD: Intervertebral disc
LPL: Lipoprotein lipase
MRI: Magnetic resonance imaging
NP: Nucleus pulposus
NP-HIF-1α KO: NP-specific HIF-1α-deficient
PPAR-γ: Peroxisome proliferator-activated receptor –γ
RUNX2: Runt-related transcription factor 2
SHH: Sonic hedgehog
VHL: Von hippel-lindau
